# Supplementary figures and images for: Transient Ascaris suum larval migration induces intractable chronic pulmonary disease and anemia in mice
Source: PLoS Negl Trop Dis. 2021 Dec 16;15(12):e0010050. doi: 10.1371/journal.pntd.0010050 (PMC8717995; doi:10.1371/journal.pntd.0010050)

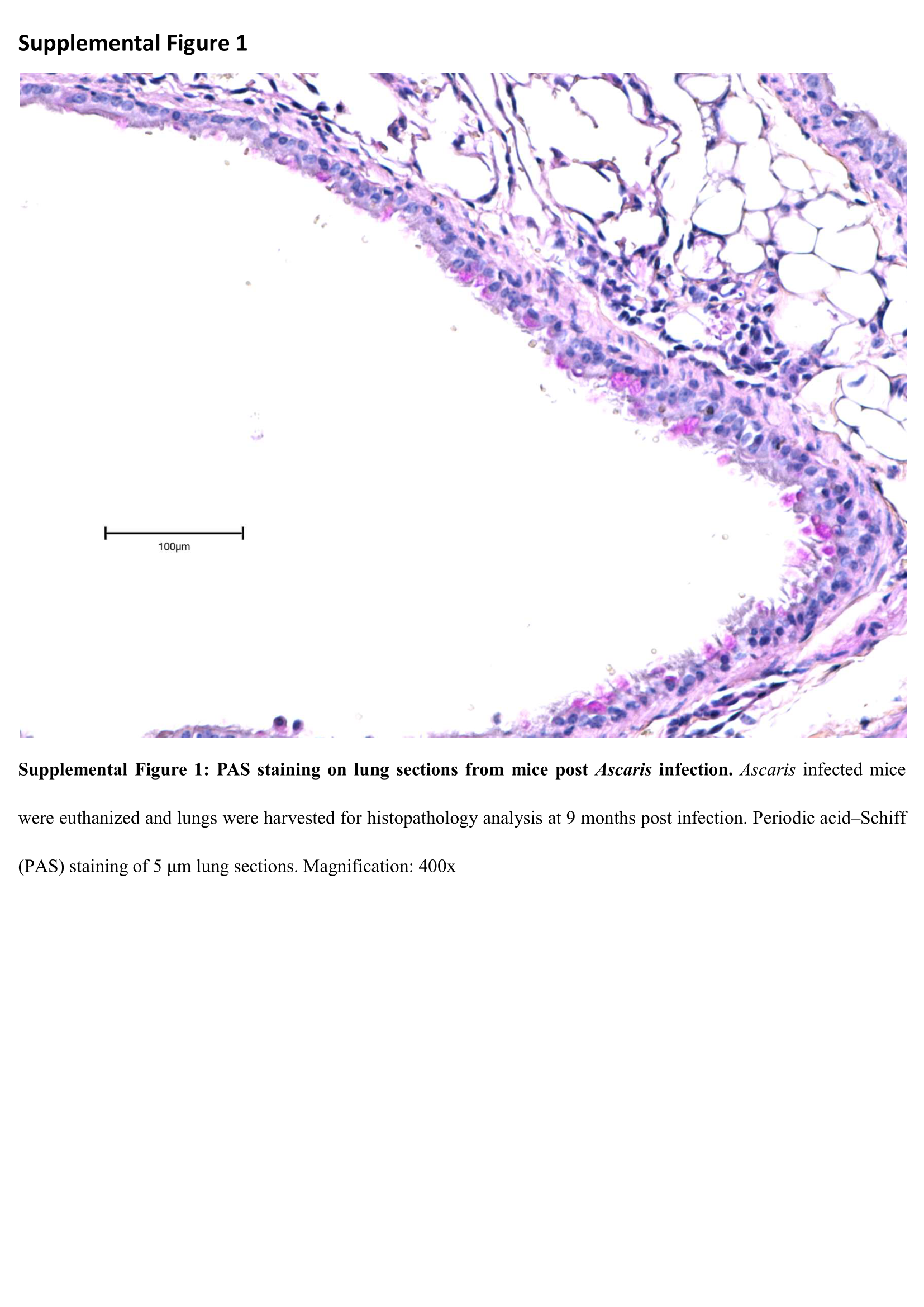

Supplement: S1 Fig — Ascaris infected mice were euthanized and lungs were harvested for histopathology analysis at 9 months post infection. Periodic acid–Schiff. (PAS) staining of 5 μm lung sections. Magnification: 400x (TIFF) [file pntd.0010050.s001.tiff]

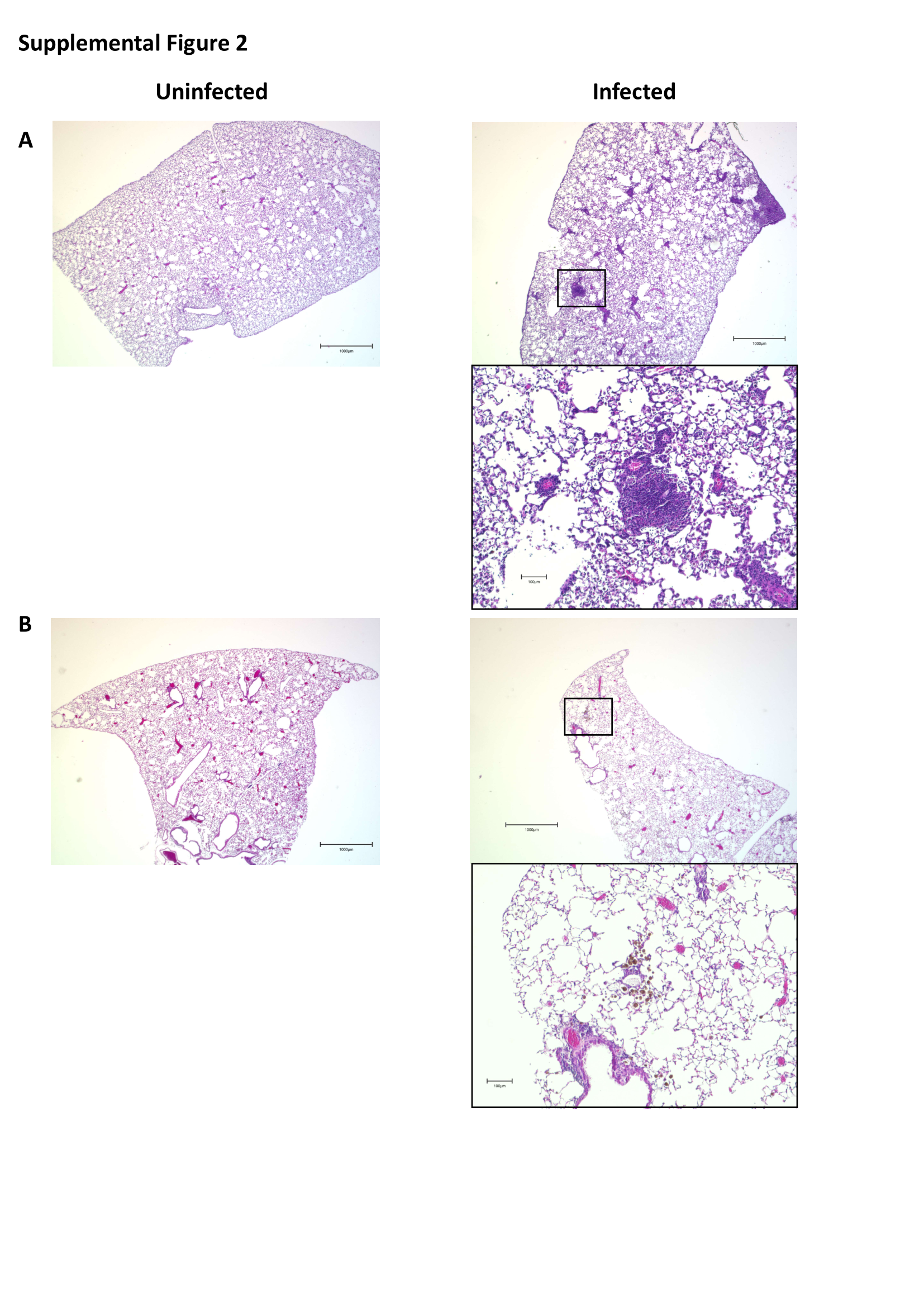

Supplement: S2 Fig — Ascaris infected mice were euthanized and lungs were harvested for histopathology analysis at (A) 12 days or (B) 9 months post infection. Haematoxylin and eosin (H&E) staining of 5 μm lung sections. Black box demonstrates area of (A) dense immune cell infiltrate and (B) hemosiderin-laden macrophages around bronchovascular bundles. Magnification: 20x and 200x. (TIFF) [file pntd.0010050.s002.tiff]

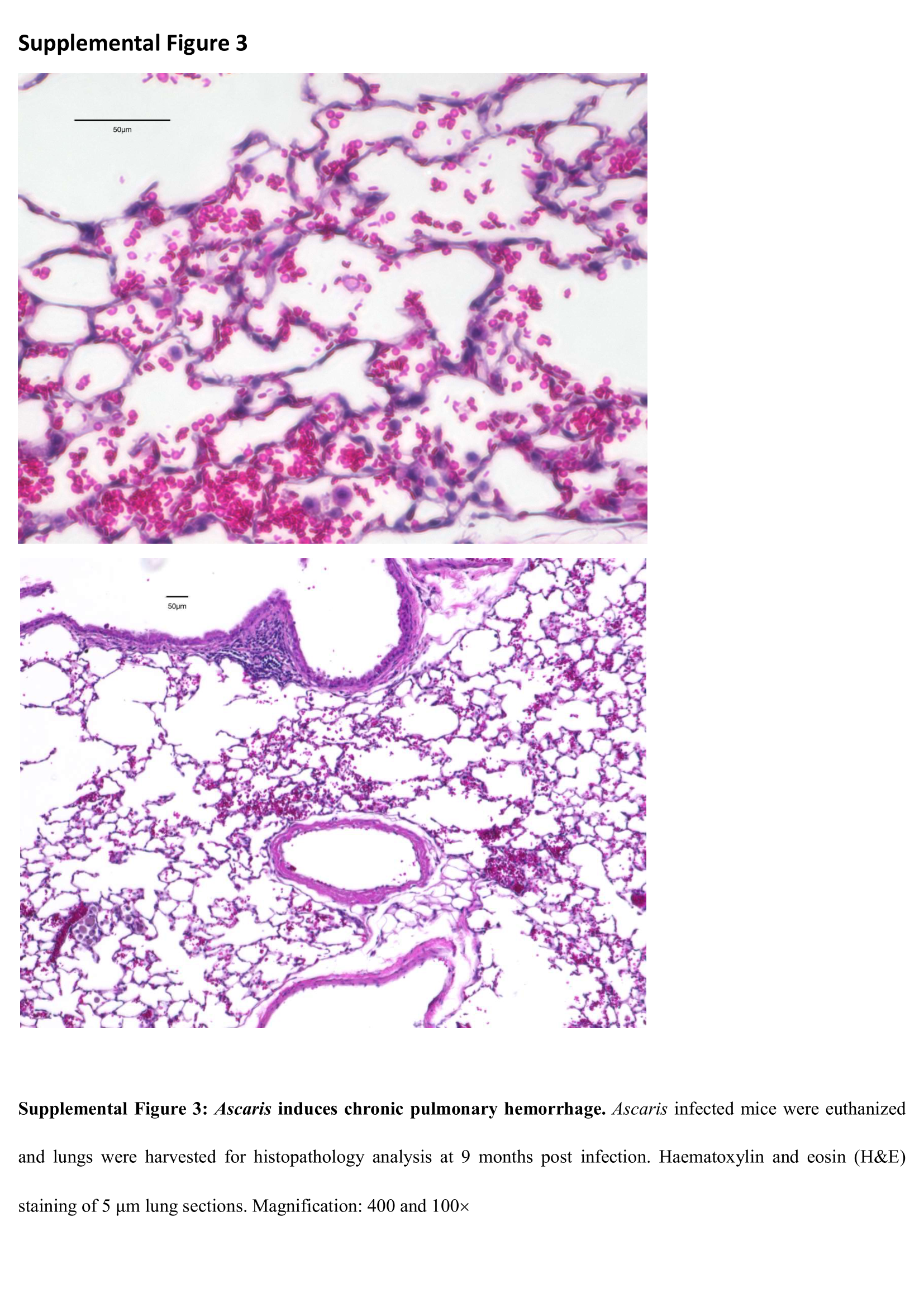

Supplement: S3 Fig — Ascaris infected mice were euthanized and lungs were harvested for histopathology analysis at 9 months post infection. Haematoxylin and eosin (H&E) staining of 5 μm lung sections. Magnification: 400x and 100x (TIFF) [file pntd.0010050.s003.tiff]

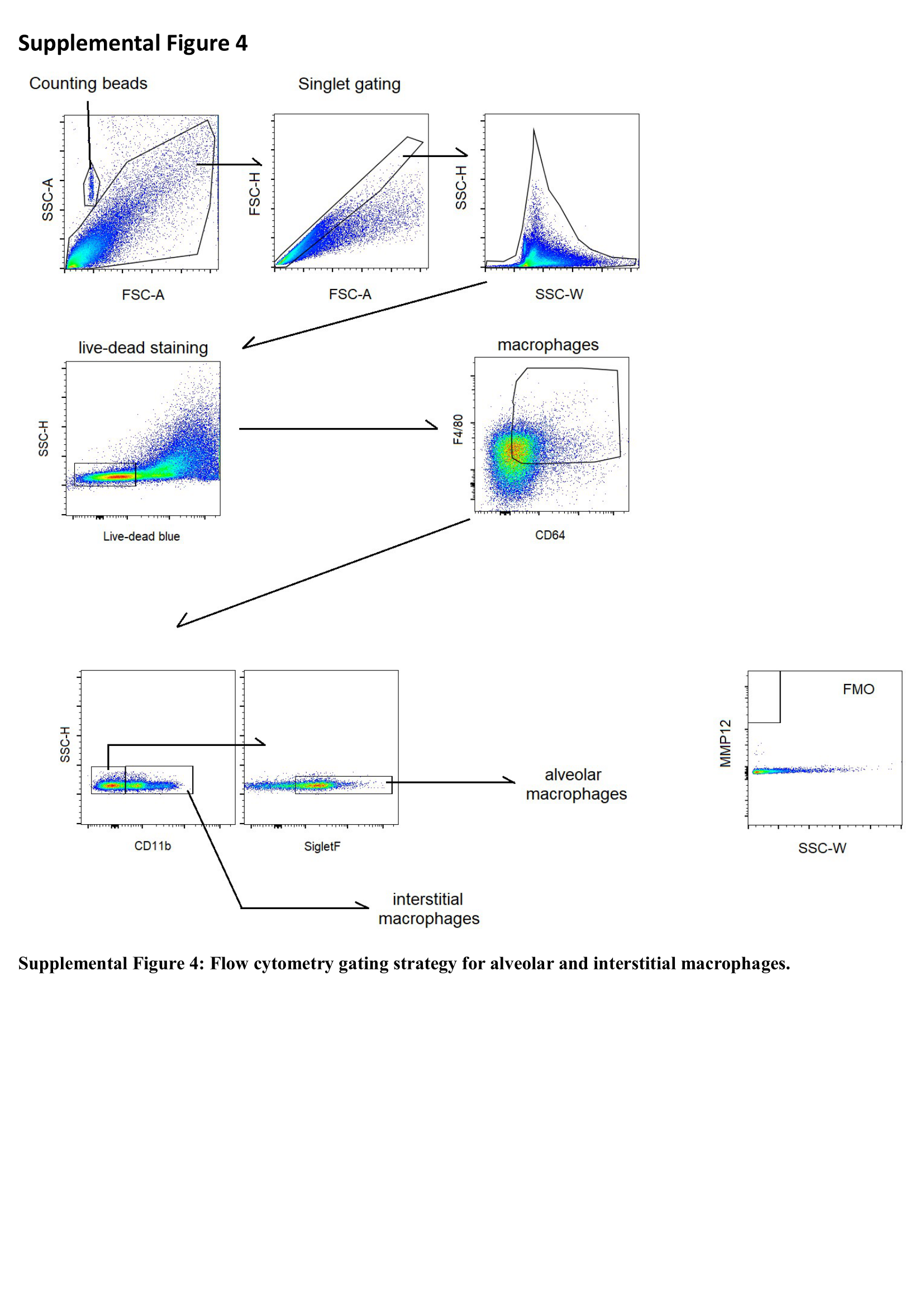

Supplement: S4 Fig — (TIFF) [file pntd.0010050.s004.tiff]
