# Supplementary material for: Transient Ascaris suum larval migration induces intractable chronic pulmonary disease and anemia in mice
Source: PLoS Negl Trop Dis. 2021 Dec 16;15(12):e0010050. doi: 10.1371/journal.pntd.0010050 (PMC8717995; doi:10.1371/journal.pntd.0010050)
Supplement: Attachment — Submitted filename: Plos Pathogens Response to Reviewers Final.docx [file pntd.0010050.s005.docx]

General: The reviewers were both positive about the study - mentioning that the experiments were well-executed and the manuscript was well-written, but raised concerns about it being descriptive and an extension from prior studies with another mouse model. Based on the suggestions, recommendation is to provide more experiments functionally investigating MMP12 and further evaluation of macrophages.

*Authors response: We want to thank the reviewers and editors for their reviews. We appreciate that the reviewers recognized the integrity of the manuscript and that this study was “the next logical step” as well as highlighted areas of improvement. Given the time course of our novel animal model (9 months) we addressed all the reviewers comments that were feasible to address within the 60 day turn-around time requested by the editors.*

**Part I - Summary**
Please use this section to discuss strengths/weaknesses of study, novelty/significance, general execution and scholarship.

Reviewer #1: Title: Transient Ascaris suum larval migration induces intractable chronic pulmonary disease and anemia in mice

Summary of Paper: This study looks at the long-term consequences of Ascaris suum infection in mice. Previous work by this group has shown that A. suum infection leads to sustained allergic airway disease well after the larvae have excited the lung. The current manuscript takes the next logical step and explores whether A. suum infection can induce other pulmonary lung conditions outside of allergic airway disease. Indeed, similar to what has been shown in the context of a mouse model of human hookworm infection, A. suum infection promoted chronic airway disease resembling emphysema. The emphysema-like phenotype persisted well after helminth clearance. In addition to the chronic lung obstruction, these mice exhibited long-term vascular leakage and as a result of A. sum driven pulmonary hemorrhage. Importantly, these persistent changes to the lung architecture (both air spaces and vasculature) also correlated with chronic anemia. This model suggests that Ascaris infections may contribute to COPD-like disease in areas endemic to helminth infections and helps to provide a context for how a single infectious episode in humans may contribute to chronic anemia.

Comments to Authors: This is a well written paper that explores an important question and provides novel insight into the long-term pulmonary consequences of helminth infection. The data is presented in a clear and logical fashion. The discussion nicely highlights the importance of these findings. Overall, this work is a nice complement to previous AHR studies by his group using their A. suum infection model. Looking at whether A. suum can also lead to long-term obstructive lung disease is an interesting next step in this research. The significance is clear as the role of helminths in the induction of other pulmonary disorders outside of allergic lung inflammation remains a black box. Importantly, the findings provided here corroborate observations in a different helminth model adding weight to the generality and importance of helminth-induced COPD/emphysema. The manuscript also extends observations looking at persistent vascular hemorrhage and chronic anemia and how such a correlation might be significant in human disease. Overall, this work has important ramifications with how we view pulmonary disease, helminth infections, and anemia in areas endemic to helminth infections. The authors may consider a few things to further increase the impact of their findings.

*Author Response: We appreciate that the reviewer recognized this current manuscript complements our previous work and, more importantly, highlights the significant public health implications of this manuscript, particularly in light of the United Nations Sustainable Development Goals 3.4, which aims to reduce by 1/3^rd^ pre-mature mortality from non-communicable diseases like chronic pulmonary disease. In order to achieve this goal it is critical to understand the causes of these non-communicable diseases before they can be prevented. Understanding the role of helminths in driving chronic lung disease in helminth-endemic regions is imperative to reaching this goal. We have also taken into consideration the thoughtful additions provided by the reviewer to strengthen the manuscript.*

Reviewer #2: This manuscript presents a descriptive overview of the long-term lung pathological consequences to Ascaris infection in mice and nicely extends the authors previously published work in this area. This work shows that a transient migration of Ascaris larval through the lung results in enhanced type 2 responses, eosinophilia, increased airway hyper-responsiveness and pathology changes such as emphysema-like damage and increased vascular leakage in the lung, effects that occur long after larvae have left the tissue or even the host. The authors propose that MMP12 production by alveolar macrophages may be responsible for the long term lung pathology.
Whilst on the whole, the experiments have been well executed and these findings do present an interesting, broad and important implication for helminth infections and global health, the findings are similar to what has been extensively studied in terms of Nippostrongylus brasiliensis infection, as the authors make a point to discuss. Currently, without exploring some of the more interesting findings, this manuscript doesn't make a significantly big enough advancement of knowledge in the field.

*Author Response: We agree with the reviewer that several aspects of this manuscript are descriptive in nature, establishing a novel chronic animal model of ascariasis and using diverse technologies (uCT, airway physiology, histology and flow cytometry) to provide a comprehensive lung disease phenotype that will support future mechanistic evaluation. We thank the reviewer for stating the experiments are well executed and that the findings present important implication for helminth infections and global health.*

*As mentioned by the reviewer the N. brasiliensis model demonstrated chronic lung disease by histology and airway hyperreactivity similar to our findings. However, because N. brasiliensis and Ascaris spp. are different species with different life cycles (i.e., time spent in the lungs, larval development within the lungs) finding similar histologic appearance and airway hyperreactivity is additive to gaining greater understanding of the role of distinct helminths in non-communicable diseases like chronic lung disease. Additionally, despite the N. brasiliensis study, helminths are still not recognized as a driving factor in the development of chronic lung disease and are still used in human clinical trials to treat non-communicable lung diseases like asthma. Our manuscript therefore acknowledges and extends the work of Marsland, et al., adding to the corpus of scientific data that support the concept that multiple helminths lead to chronic lung damage. We believe our findings are critical for defining the rationale to alter public health measures with the goal of reducing the high rates of chronic lung and related diseases in parasite endemic regions globally.*

*However, there are several additional differences between our study and that of Marsland, et al., that provide significant novelty. First, our findings show for the first time more complete lung physiologic characterization, specifically compliance measurements, that have not previously been reported. Lung compliance is a cornerstone measurement of the chronic lung disease phenotype; moreover CT imaging provided additional novel information regarding lung size that was not previously assessed. Use of these advanced technologies thus informs specifically on functional and structural impact of larval migration through the mouse lungs that have never previously been reported. Second, our manuscript demonstrates the impact of larval migration on the development of anemia, which has also not previously been shown.* *It is classically believed that severe anemia in parasitic-endemic regions is a result of gastrointestinal parasites and malnutrition, but our manuscript uncovers the novel finding that anemia can be caused by isolated helminth-induced lung disease. This is a paradigm shifting finding that paves the way for substantial additional preclinical and human studies.*

**Part II – Major Issues: Key Experiments Required for Acceptance**
Please use this section to detail the key new experiments or modifications of existing experiments that should be absolutely required to validate study conclusions.

Generally, there should be no more than 3 such required experiments or major modifications for a "Major Revision" recommendation. If more than 3 experiments are necessary to validate the study conclusions, then you are encouraged to recommend "Reject".

Reviewer #1: 1) Given the focus on MMP12 and its potential correlation with COPD, there are some avenues to explore. However, do the authors believe that MMP12 driving A. suum-induced COPD/emphysema? MMP12 KO mice did not show a difference in emphysema-like lung disease compared to wildtype mice in the N. brasilensis model of COPD/emphysema. Is there something more likely that could be tested to inform mechanism? At the very least, some discussion of this in the manuscript would seem important.

*Author Response:* *Due to the known role of proteases in the development of chronic lung disease (MMP-12, MMP-9 and Neutrophil Elastase) we hypothesize that the high levels of MMP-12 likely contributed to the chronic lung phenotype that developed as MMP-12 upregulation was not seen in our naïve mice. Studies evaluating the impact of Ascaris infection in MMP-12 KO mice will be critical for testing this hypothesis and is part of our future studies. The Marsland, et al., study did use MMP-12 KO mice, however the only end point evaluated was histology at day 30 post-infection; no quantitative, functional or immunologic evaluation was completed. Based solely on one histologic image at one time period post-infection, it can’t be concluded that MMP-12 is not required for the development of chronic lung disease. For example, it is likely (as seen in the data presented in this manuscript) that upregulation of neutrophil elastase early in disease may influence the development of the emphysematous phenotypes acutely and, because of chronically upregulated MMP-12 expression, MMP-12 may play a role acutely and chronically. If this were the case, using MMP-12 KO mice we may see an emphysematous phenotype acutely at day 30 post-infection due to neutrophil elastase however the phenotype resolves chronically in the absence of MMP-12. Furthermore, investigations with longitudinal, quantitative assays are needed as applied to our model. However, as a single experiment our model requires 9 months to complete, the studies are unable to be completed and incorporated into this manuscript in the appropriate timeframe. As suggested by the reviewer, a more thorough discussion of this was introduced into the manuscript (Line 303-310)*

2) There is evidence that the amount of Ascaris eggs provided during the initial infection can have an impact on the health of the animal (ex: see Lewis et al. J Helminthol. 2009 Mar;83(1):1-5.). Because data from N. brasiliensis-driven COPD-like disease might suggest that damage caused by worms is the driver of the persistent COPD-like phenotype, would titration of the eggs (which would translate into less damage at lower dose due to fewer larvae in the lungs) be informative? Does phenotype of persistent vascular leak and emphysema only occur at high dose infection? Do you see the same phenotype at 100 eggs, 500 eggs? This should at least be considered in the discussion if not provided experimentally.

*Author Response: We are in agreement with the reviewer that the amount of Ascaris eggs impacts the health and phenotype of the animal as well as in humans. The literature standardized 2500 Ascaris eggs used in mouse models of ascariasis have been established to effectively develop the equivalent of human eosinophilic pneumonitis, a classic acute complication of pulmonary larval migration in humans. This dose was determined by our colleagues and co-authors of this paper, Dr. Ricardo Fujiwara and team, through titration experiments. Given smaller boluses of Ascaris eggs correlates with less mechanical destruction and less immune activation leading to less severe eosinophilic pneumonitis and allergic airway disease, we assume this would hold true for persistent vascular leak and emphysema. As suggested by the reviewer more discussion was added into the methods and discussion (Line 252-253; 332; 360-364). We have also added in the reference provided by the author supporting this is likely dose-dependent.*

Reviewer #2: 1. The finding that MMP12 is increased in the lungs of infected mice, fits nicely with the observation that there is increased emphysema-like damage. However, this has been described (as the authors point out) for Nippostrongylus infection (Marsland et al). What is the consequence of MMP12 beyond pure observational correlation to pathology? During Nippo infection, MMP12 KO mice still develop the same pathology and emphysema-like damage as a wild-type mouse, begging the question of whether the damage by Ascaris is MMP12 dependent? If so, it would be a major advance and an extremely interesting and novel finding in light of work with Nippostrongylus and COPD and would be worth exploring. Could the authors use an MMP inhibitor or a MMP KO mouse to test this?

*Author Response: We agree with the reviewer that more information regarding the role of MMP-12 is needed although its functional role is supported by standard emphysematous mouse models as described in the manuscript. As mentioned by the reviewer, the Marsland, et al., study did use MMP-12 KO mice however the only end point evaluated was histology at day 30 post-infection; no quantitative, functional or immunologic evaluation was completed. Based solely on one histologic image at one time period post-infection, it can’t be concluded that MMP-12 is not required for the development of chronic lung disease as a result of N. brasiliensis or any other helminth infection. For example, it is likely (as seen in the data presented in this manuscript) that upregulation of neutrophil elastase early in disease may influence the development of the emphysematous phenotypes acutely and, because of chronically upregulated MMP-12 expression, MMP-12 plays a role both acutely and chronically. If this were the case, using a MMP-12 KO mouse you may see an emphysematous phenotype acutely at day 30 post-infection due to neutrophil elastase but that phenotype resolves chronically in the absence of MMP-12. Further investigation with longitudinal, quantitative assays are needed. Our model is primed to answer this very question. However, as our model is 9 months long, the studies are unable to be completed and incorporated into this manuscript in the requested timeframe. We will be working on the longitudinal and comprehensive evaluation using both MMP-12 KO as well as MMP-12 inhibitors in future studies. However, given the critical nature of the reviewer’s concerns a more thorough discussion of this concept was introduced into the manuscript (Line 303-310)*

2. Are alveolar macrophages the only important source of MMP12, as stated by the authors? What about interstitial macrophages, or epithelial cells? On the flow plots (Fig 3D, there looks to be two different populations of MMP12+ macrophages - does this reflect inflammatory macrophages as well as interstitial macs, perhaps plotting MMP12 vs CD11b (instead of SSC) would help distinguish this? If alveolar macrophages are the only important cell in terms of MMP12 activity, this could be an interesting finding and may explain differences between Nippo and Ascaris? Does the enhanced type 2 response in the lung directly relate to an increase in MMP12 levels in alveolar macrophages. Are there other M2 markers that suggest alveolar macrophages are the key responding cell that contributes to pathology in the lung?

*Author Response: MMP-12 is classically sourced from macrophages in general and not specific to alveolar macrophages. We appreciate the reviewer highlighting this miscommunication in our manuscript. We have added that interstitial macrophages also contribute to the production of MMP-12 throughout the text. We also included data into Figure 3d and 3e and in the text demonstrating that both alveolar and interstitial macrophages contribute to MMP-12. We hope the addition of interstitial macrophages helps clarify the questioned populations identified in Figure 3 by the reviewer. Both interstitial and alveolar macrophages play an inflammatory role in human chronic lung disease (Dewhurst et al. Scientific Reports. 2017; Voelkel et al. Proceedings of the American Thoracic Society. 2006). Classically, alveolar macrophages are the first cells to respond to airborne irritants (smoking) in the lungs leading to the initiation of chronic lung disease and thus play a key role in development of chronic lung disease (Vlahos et al. Frontiers in immunology. 2014). However, the mechanism of injury in Ascaris driven chronic lung disease is unconventional due to the unique larval life cycle, in that the initial insult is vascular followed by interstitial and then alveolar. Thus, interstitial macrophages may play a larger role in the development of chronic lung disease as a result of ascariasis in comparison to airway irritant derived emphysema.* *This has been added into the manuscript as suggested by the reviewer.*

3. The changes in lung pathology over time are interesting, but currently are not presented in a way that the reader can fully appreciate. The CT scans and changes in lung volume and measurements of MLI are great inclusions in Fig 2. However, as the authors state, larval migration is not consistent and there will be variation across a lung. Therefore, low magnification H&E images of the entire time course (especially in light of no measurements of MLI at early time points), need to be shown comparing naive and infected animals. The authors show increased Muc5ac mRNA expression, but does this relate to increased goblet cell hyperplasia which may relate to increase type 2 signature and allow the authors to conclude a type 2 airway pathology exists?

*Author Response: We agree with the reviewer and have added low magnification H&E images of the time course to compare PBS, naïve lungs histology and Ascaris infected lung histology at day 12 post-infection as well as 9 months post-infection in order to highlight the areas where MLI measurements were assessed along the bronchoalveolar regions (supplemental figure 2). Goblet cell hyperplasia at day 12 post-infection using PAS was demonstrated in our previous paper (Weatherhead et al. Infection and Immunity. 2018) however we have included a representative images of goblet cell hyperplasia using PAS stain at 9 months into the supplemental images to support the findings regarding increased Muc5ac mRNA expression as recommended by the reviewer (supplemental figure 1).*

**Part III – Minor Issues: Editorial and Data Presentation Modifications**
Please use this section for editorial suggestions as well as relatively minor modifications of existing data that would enhance clarity.

Reviewer #1: 1) MMP12 correlated with alternatively activated macrophages in the N. brasiliensis model. Is this true in the Ascaris model?

*Author response:* *This is an interesting question posed by the reviewer. Unfortunately further phenotyping of the macrophages was not evaluated in this study. It is assumed that due to the persistent type-2 response (noted by IL-4, IL-5, IL-13 upregulated through 3 months post infection identified in this manuscript as well as known persistent elevated Th2 cells chronically in Ascaris infected mice which is data not included in this manuscript) would preferentially influence the macrophages towards the AAM phenotype. However, further analysis is needed to confirm this.*

2) The infectious dose used is 2500 eggs. The authors state that this is the accepted dose in mouse models (“…has been standardized in the literature in order to replicate human disease in a mouse model.”). However, it is unclear from references provided how this relates to human Ascaris load. Authors should define more in the text (not just in methods).

*Author response:* *In agreement with the reviewer more detailed text was included to demonstrate the Ascaris infectious dose relevance to human disease (line 252-253; 361-364).*

3) While cytokine and chemokine expression were investigated after in vitro culture of cells obtained from digested lung, this does identify the cells responsible for their production. Unclear how this adds much over bulk mRNA analysis of total lung. A more in depth look at the cells involved might increase impact or provide more clarity.

*Author Response:* *Type-2 cytokines and chemokines were included in the analysis to indicate the evolution of type-2 inflammatory longitudinal changes occurring in the lungs. However, the objective of the manuscript is not focused on changes in cytokine and chemokine expression thus longitudinal bulk transcriptomic work is outside the scope of this paper and expression of specific targets (determined from our previous work) was used instead.*

4) The manuscript often refers to helminth infection as driving allergic inflammation. Is this the right terminology as no allergen is provided? Would type-2 inflammation be more accurate?

*Author Response: We appreciate this feedback by the reviewer. While ascariasis does serve as an allergen in early disease to cause allergic airway disease, in this study we do not show IgE upregulation to support the definition of allergic inflammation. Thus in agreement with the reviewer “allergic inflammation” was changed to “type-2 inflammation” where indicated.*

5) Figure 2D is a little confusing. Why is lung compliance in the naïve mice increasing over time? How do these values compare to mouse models of smoking-induced COPD?

*Author Response:* *We appreciate the reviewer highlighting this area of confusion. The measurement of compliance as outlined in the methods is based on total lung capacity at a standard increased lung pressure. Thus as mice age and grown, their lungs also get larger and as a result of larger lungs at baseline their total lung capacity also get bigger at baseline. Compliance in mouse models of smoking-induced emphysema, mouse models of elastase-induced emphysema as well as mouse models of genetic spontaneously developing emphysema have been shown to be increased. Additionally, compliance is known to increase in human smoking-induced emphysema.*

6) Figure 3D is an important finding as it corroborates the mRNA data. However, given infection can increase autofluorescence among macrophages in the lung, it would seem an isotype control would provide more rigor to these conclusions.

*Author Response: We understand the reviewers concern regarding autofluorescence of lung cells and for that reason have chosen AF647 for MMP-12 as the final readout for the least amount of autofluorescence via the red laser (excite wavelength 633nm). Minimal autofluorescence were observed on the FMO on MMP-12. In recent studies, isotype controls are no longer considered “standard negative” as previously thought as they poorly control for autofluorescence and have been proven to have great limitations to control for non-specific background staining (Hulspas et al. Cytometry Part B 76B: 355-364. 2009; Maecker and Trotter. Cytometry Part A 69A: 1037-1042. 2006). Per Hulspas et al states “The potentially different nonspecific binding characteristics of an isotype antibody, the possible pitfalls in the interpretation of the data and the notion that it is virtually impossible to manufacture the perfectly matched isotype antibody, have lead to the reconsideration of earlier recommendations. Over the last decade or so, it has become more widely accepted that isotype controls are of little value in distinguishing positive from negative and should therefore not be used to set positive gating regions for test antibodies. Isotype controls are not recommended for use in quantitative (cell counting) assays to determine nonspecific binding. ”*

Reviewer #2: 1. The authors suggest that larval migration leads to allergic airway disease phenotype, but I'm not sure what the authors mean by "allergic" here. Rather I think it would be more accurate to state that larval migration induces type 2 immune pathology in the lung or something similar. The data described in no way suggests an "allergic" response.

*Author Response:* *As we do not show IgE responses to support the allergic airway disease phenotype we have adjusted the wording as suggested by the reviewer to “type-2 immune response” and “type-2 immune pathology”. We appreciate the reviewer highlighting this wording error.*

2. It was a little unclear for some figures whether the naive group of mice represent a group of different aged mice (up to 9 months)? In fig 2, this is nicely shown with naive mice at each time point, but for other figures, this isn't clear. If not what age are the naive mice and considering the extensive time course, could age impact these findings

*Author Response: We appreciate the reviewer bringing this to our attention. To clarify, age-matched naïve control mice were used for all experiments at all time periods (as described in the methods section Line 364-366).* *Given the complexity of Figure 1 we show only one set of naïve samples per graph as the values for the naïve control mice were consistent across all time periods*. *This has been updated and clarified in the figure legend.*

3. Sup Figure showing macrophage gating doesn't have all the information needed that is required to understanding the gating - for instance are the lower flow plots just showing the alveolar macrophages? How did the authors decide on MMP12 positive gate - this is very unclear from the figure/methods

*Author response: We appreciate the reviewer bringing this to our attention and have added additional information regarding our gating strategy to the supplemental Figure 4* *and have included gating for interstitial macrophages.*

4. Scale bars appear to be missing from fig 4A

*Author response: Scale bars were added to Figure 4A*

5. Data for the serial-block EM needs further explanation for readers not familiar with these types of images. It is unclear of how these images reflect edema and extravasation especially compared to what a healthy lung would look like?

*Author response: We have updated the figure with the serial-block EM reconstruction to note the intravascular space, they type 1 and type 2 pneumocytes as well as the RBC extravasating in between the type and type 2 pneumocytes into the alveolar space in figure 4H.*

6. Methods have good details provided in general, just some missing information in relation to how or if mRNA measurements were normalised to a housekeeping gene etc?

*Author response: We have added that the mRNA measurements were normalized to the 18s housekeeping gene in the methods.*

7. Figure legends need a little more information for a reader to fully understand the details presented including what some abbreviations stand for (eg Fig 2D - TLC. Fig 3 D-E it would help to have the time point of infection

*Author response: Thank you to the reviewer for identifying the lack of sufficient information in the figure legends. All figure legends have been expanded and abbreviations have been explained in the figure legends.*
