# Supplementary material for: Transient Ascaris suum larval migration induces intractable chronic pulmonary disease and anemia in mice
Source: PLoS Negl Trop Dis. 2021 Dec 16;15(12):e0010050. doi: 10.1371/journal.pntd.0010050 (PMC8717995; doi:10.1371/journal.pntd.0010050)
Supplement: Attachment — Submitted filename: PLOS NTD Cover Letter re-submission.docx [file pntd.0010050.s006.docx]

**National School of Tropical Medicine**

One Baylor Plaza, BCM113

Houston, TX 77030-3411

713-798-1199

Fax 713-798-2299

November 23, 2021

Dear Dr. Keke C Fairfax,

Attached please find our revised manuscript entitled “Transient *Ascaris suum* larval migration induces intractable chronic pulmonary disease and anemia in mice” (PNTD-D-21-015). The manuscript is being resubmitted for consideration in PLOS Neglected Tropical Diseases as a research article.

We want to thank the reviewers and editor for their thoughtful comments. We have added the three articles into the introduction and discussion sections as well as added the number of mice per group into the methods as requested by the reviewers. We believe the edits made, based on the reviewer and editor comments, have strengthened this manuscript. Furthermore, we believe that this updated manuscript will provide the readers of PLOS NTDs with new insight into parasite-host interactions suggesting parasites like ascariasis are driving non-communicable diseases in low- and middle-income countries (LCIM). This important data will ultimately shift the paradigm on our understanding of how microbes influence non-communicable diseases particularly in LMIC. These findings have significant implications for informing public policy and shaping strategies to urgently reduce global parasite-induced morbidity.

All authors have seen and approved the manuscript, it has not been previously published or being considered for publication elsewhere. The submitted work is original. The authors report no conflicts of interests and have participated in the study and concur with the submission.

Thank you for your consideration,

Jill E. Weatherhead, MD, PhD, CTropMed

Assistant Professor

Director of Medical Education, National School of Tropical Medicine

Department of Pediatrics, Sections of Tropical Medicine and Infectious Diseases

Department of Medicine, Section of Infectious Diseases

Baylor College of Medicine

Houston, TX, USA
